# Eosinophilic Liver Abscess: A Case of Locally Acquired *Fasciola hepatica* in Alberta, Canada

**DOI:** 10.1093/ofid/ofae101

**Published:** 2024-02-19

**Authors:** Teagan King, Miguel M Cabada, Kinga Kowalewska-Grochowska, Stephen D Vaughan

**Affiliations:** University of Calgary, Department of Medicine, Division of Infectious Diseases, Calgary, Alberta, Canada; University of Texas Medical Branch, Department of Medicine, Division of Infectious Diseases, Galveston, Texas, USA; Universidad Peruana Cayetano Heredia, Department of Medicine, Division of Infectious Diseases, Lima, Peru; University of Alberta, Department of Pathology, Division of Microbiology, Edmonton, Alberta, Canada; University of Calgary, Department of Medicine, Division of Infectious Diseases, Calgary, Alberta, Canada

**Keywords:** Alberta, Canada, *Fasciola hepatica*, hepatic abscess

## Abstract

*Fasciola hepatica* is a trematode causing acute and chronic infection. A 33-year-old Canadian woman with eosinophilic liver abscesses and no relevant travel was diagnosed with *F hepatica* infection*. F hepatica* is reported in livestock in Alberta. This is the first case of locally acquired fascioliasis in Canada in >100 years.

*Fasciola hepatica* is a liver trematode with worldwide animal distribution, most commonly seen in sheep- and cattle-raising areas [1, 2]. Imported human *Fasciola* infections have been reported in the United States and Canada [2–9], with only a few cases of local transmission documented in the United States [10–12] and possibly 1 remote case in Canada [13]. We describe a case of locally acquired *F hepatica* infection in Alberta.

## CASE REVIEW

A 33-year-old woman presented with 3 weeks of progressive epigastric and right upper quadrant pain. Abdominal ultrasound revealed mass-like liver lesions. The patient had no travel outside of North America, visiting Hawai’i 10 years prior and California 5 years prior. Her diet included store-bought sushi, cooked meat, and imported bagged salad. She denied eating vegetables from a local house garden and farmer's market or consuming wild vegetables. Her medical history was significant for a benign mediastinal fibrotic tumor, asthma, and eczema. She was well appearing with diaphoresis, and examination demonstrated tenderness in the epigastric region and right upper quadrant.

**Figure 1.**
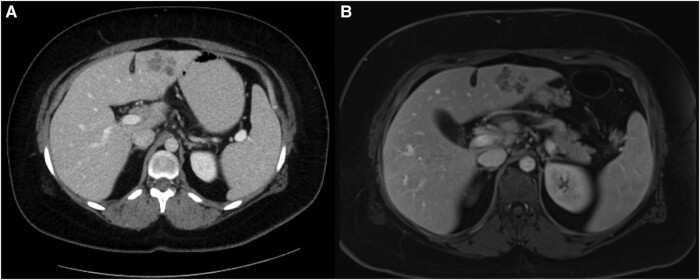
*A*, Hepatic abscesses on abdominal axial view of computed tomography show multiple small abscesses in segment 2/3. *B*, Hepatic abscesses on T1-weighted magnetic resonance imaging axial view measure 2.9 × 3.6 cm in segment 3.

Investigations revealed eosinophilia of 1.8 × 10^9^/L (reference range, 0.0–0.7 × 10^9^/L), noting that her eosinophils were within range 3 months prior. Computed tomography of the abdomen and pelvis demonstrated multiple small hepatic abscesses (Figure 1*A*).

Abscess aspiration was performed with minimal drainage. The bacterial, fungal, and *Mycobacterial* culture results were negative. Results were also negative from multiple stool ova and parasite examinations and bacterial stool reverse transcriptase polymerase chain reaction (PCR) plus culture.

Magnetic resonance imaging was performed (Figure 1*B*), with liver biopsy revealing eosinophilic infiltration, negative bacterial culture, and negative tissue PCR for *Echinococcus*. The serology result was negative for *Entamoeba histolytica*, *Coccidioides*, *Histoplasma*, *Strongyloides*, *Toxocara*, *Echinococcus multilocularis*, *Echinococcus granulosus*, and *Coxiella burnetii*. The *F hepatica*–specific serology performed by indirect enzyme immunoassay (in house) at the National Reference Centre for Parasitology in Montreal, Canada, was strongly positive, with a sample optical density of 1.50 (reference range: negative <0.40, equivocal = 0.40–0.49, positive ≥0.50).

Repeat stool ova and parasite investigations were negative for *Fasciola* eggs. A stool DNA sample sent to the Cusco Branch of the Tropical Medicine Institute of Universidad Peruana Cayetano Heredia in Peru for *F hepatica* real-time PCR [14] was positive, confirming the diagnosis of fascioliasis. The patient received triclabendazole (750 mg, 10 mg/kg) orally every 12 hours for 2 doses and had complete symptomatic and biochemical (eosinophilic) resolution.

## DISCUSSION

### Microbiology and Life Cycle

*Fasciola* species are flat trematodes, also known as liver flukes, that infect the biliary tree of humans and livestock [1, 2]. *F hepatica* and *F gigantica* are the only 2 species of *Fasciola* known to infect humans [1, 2], the latter restricted to Africa and Asia. Eggs excreted in the feces from the definitive host (eg, ruminants) embryonate in fresh water and hatch into miracidia, infecting lymnaeid freshwater snail intermediate hosts [1, 2]. After asexual reproduction in the snail, free-swimming cercariae are released and transform into metacercariae that encyst on aquatic vegetation, commonly watercress. Once ingested by a new host, including humans, metacercaria transform into a juvenile fluke that penetrates the intestinal wall and migrates through the liver parenchyma to the biliary tree. Mature flukes develop, releasing eggs excreted in feces and completing the life cycle.

### Clinical Presentation

The 2 phases of infection—acute (migratory) and chronic (biliary)—reflect the parasite location and stage. The acute phase causes fever, right upper quadrant pain, nausea, vomiting, weight loss, jaundice, abnormal liver enzymes, eosinophilia, and hepatomegaly due to migration of immature flukes through liver parenchyma [1]. The chronic phase causes biliary colic, cholecystitis, cholangitis, or pancreatitis due to adult flukes causing obstruction in the biliary tree [1–3]. Most *F hepatica* infections in endemic areas are asymptomatic or subclinical. As our patient presented acutely with right upper quadrant pain (signifying infection in the last few months) and eosinophilia, her exposure was unlikely travel related, which was >5 years prior.

### Diagnosis

Fascioliasis can be suspected clinically in highly endemic areas and can be confirmed acutely with serology, as the stool microscopy result will be negative until mature flukes migrate to the bile ducts and release eggs (chronic phase). The chronic phase is diagnosed with direct visualization of eggs on stool microscopy and/or serology. Our patient had a clinical presentation compatible with acute fascioliasis and highly positive serology. We confirmed the diagnosis with stool PCR (after negative stool microscopy findings). Stool PCR sensitivity is greater than that of microscopy, and experimental infection in animal models has shown that stool PCR detection is positive weeks prior to visualization of eggs on microscopy [15, 16].

### Treatment

The only recommended treatment for acute or chronic stages of infection is triclabendazole (10 mg/kg) every 12 hours for 2 doses [1]. Access to triclabendazole in Canada is limited to special authorization.

### Epidemiology

*F hepatica* is endemic in animals in the Americas, Europe, Asia, and Africa [1, 2]. In North America, fascioliasis is endemic in Mexico, with a few locally acquired cases from Hawai’i [4, 12], Florida [10], and California [4, 11]. There is 1 case of possible locally acquired human fascioliasis in Canada [13], with just 5 published reports of imported cases [5–10]. The only previous report of human fascioliasis possibly acquired in Canada contains limited details of the patient’s presentation and no details of travel history, but autopsy performed due to meningitis (no details provided of the etiology; eg, bacterial vs parasitic) confirmed *F hepatica* fluke in the bile duct of the liver in an Italian immigrant in 1905 [13].

*F hepatica* was reported in Alberta beef cattle in 1998 [17] and British Columbia dating back to 1916 [18]. Agriculture Alberta has detected the presence of *F hepatica* in farmed wapiti (elk), bison, cattle, and sheep within the province [19–21], while others have documented *F hepatica* within dairy cows in Québec [22]. These reports confirm that the life cycle of fascioliasis is supported in Canada and that theoretical risk of infection to humans is present if eating wild encysted vegetables (eg, watercress). With environmental changes relating to climate change, it is likely that human fascioliasis cases will occur in new areas where it has not previously been identified [23], such as Canada, thus requiring a high index of suspicion to timely diagnose.

Our patient denied the consumption of local wild vegetables, including lettuce and watercress. In this case, transmission possibly occurred through consumption of watercress or lettuce grown in an endemic area that was then imported in bagged salad. The lack of travel history and lack of local knowledge regarding livestock *Fasciola* epidemiology resulted in delayed diagnosis, which has been reported in Argentina [24].

## CONCLUSION

Fascioliasis can be acquired in nonendemic areas in patients without a travel history, and the diagnosis requires a high index of suspicion. Patients with unexplained liver lesions and eosinophilia should be evaluated for fascioliasis in the absence of travel or endorsed consumption of vegetables able to encyst *Fasciola*, such as lettuce and watercress.

## Notes

***Acknowledgments.*** Hong Zhou, MD, Provincial Laboratory of Alberta.

***Author contributions.*** T. K.: writing–original draft, writing–review and editing, visualization, project administration. M. M. C.: conceptualization, writing–review and editing, resources. K. K.-G.: conceptualization, writing–review and editing. S. V.: conceptualization, writing–review and editing.

***Patient consent statement.*** Patient's consent obtained. The design of this work does not require local ethics approval.
